# Prognostic Significance of the Systemic Immune-Inflammation Index in Patients With Cholangiocarcinoma: A Meta-Analysis

**DOI:** 10.3389/fonc.2022.938549

**Published:** 2022-07-07

**Authors:** Xue-chun Liu, Yue-ping Jiang, Xue-guo Sun, Jian-jian Zhao, Ling-yun Zhang, Xue Jing

**Affiliations:** Department of Gastroenterology, The Affiliated hospital of Qingdao University, Qingdao, China

**Keywords:** cholangiocarcinoma (CCA), systemic immune-inflammation index, prognosis, overall survival, meta-analysis

## Abstract

**Background:**

The systemic immune-inflammation index (SII) is a significant prognostic factor for neoplastic diseases. However, the prognostic value of SII in patients with cholangiocarcinoma (CCA) remains unclear. This meta-analysis aimed to investigate the prognostic value of preoperative SII in patients with CCA.

**Method:**

We systematically searched for relevant studies in PubMed, Scopus, EMBASE, Web of Science, PROSPERO, and Cochrane Library databases up to March 22, 2022. Hazard ratios (HRs) and 95% confidence intervals (CIs) were used to estimate the association between SII and survival outcomes, including overall survival (OS) and recurrence-free survival.

**Results:**

Five studies with 1402 patients were included in this meta-analysis to determine the prognostic value of preoperative SII. The results showed that a higher SII was associated with poor OS in patients with CCA who underwent invasive surgery (HR=1.916; 95% CI, 1.566–2.343; Z=6.329; *P*<0.001). The results were reliable in the subgroup analysis according to country, age, sample size, SII cutoff values, and treatment methods.

**Conclusions:**

A high preoperative SII appears to be an effective and practical method for monitoring survival in patients with CCA.

**Systematic Review Registration:**

International Platform of Registered Systematic. Review and Meta-Analysis Protocols (INPLASY), identifier INPLASY202240015.

## Introduction

Cholangiocarcinoma (CCA) refers to a spectrum of invasive adenocarcinomas arising from the biliary tree. Some countries have shown an increasing incidence rate from 0.1 cases per 100,000 to 0.6 per 100,000 over the past 30 years (1, 2). CCA is a rare and aggressive malignancy that is usually asymptomatic in its early stages (3). Surgical resection remains the mainstay of curative therapy for CCA. However, the overall 5-year survival rate ranges from 25% to 40% after surgical resection (4, 5). Postoperative survival is unsatisfactory because of the high risk of complications, recurrence, and metastasis (6) .

Chronic inflammation has been related to various steps of oncogenesis, including cellular transformation, promotion, survival, proliferation, invasion, angiogenesis, and metastasis (7, 8). The systemic immune-inflammation index (SII), a systemic inflammatory marker based on platelet, neutrophil, and lymphocyte levels, has been associated with prognosis in patients with cancers, such as lung, hepatocellular, colorectal, and esophageal cancer (9–12). In these cancer patients, a high SII is associated with shorter overall survival (OS) (13, 14)

However, it remains unclear whether SII is related to the prognosis of CCA. Therefore, this study aimed to investigate the prognostic value of preoperative SII in patients with CCA.

## Materials and Methods

### Study Guideline and Ethnics

This meta-analysis was performed in accordance with the Preferred Reporting Items for Systematic Reviews and Meta-Analysis guidelines (15). Ethical approval was not required for this meta-analysis because the data were extracted from published studies.

### Search Strategy

We systematically searched for relevant studies in PubMed, Scopus, EMBASE, Web of Science, PROSPERO, and Cochrane Library databases up to March 22, 2022. The search terms used were as follows: (cholangiocarcinoma OR bile duct neoplasm OR bile duct cancer OR biliary tract neoplasm OR biliary tract cancer OR cholangiocellular carcinoma) AND (systemic immune-inflammation index OR systemic immune inflammatory index OR SII). Moreover, we manually verified the references of eligible articles.

### Selection Criteria

Two authors independently searched for relevant studies and screened the literature using titles and abstracts. The inclusion criteria were as follows: 1) studies investigating the relationship between SII and prognosis of CCA; 2) patients with CCA confirmed by pathological examination; 3) patients who had undergone surgery or invasive surgery; 4) available data of preoperative SII; and 5) patients divided into high and low SII groups according to a cutoff value and followed up over a period of time.

The exclusion criteria were as follows: 1) studies that did not exclude gallbladder or ampullary neoplasm; 2) unclear nonoperative treatment or therapy method; and 3) unavailable hazard ratios (HRs) and 95% confidence intervals (CIs) of survival outcomes.

### Data Extraction

Two authors collected data from the studies and resolved conflicts through discussion and consensus. The following information was extracted from these studies: first author, publication year, country, study duration, sample size, follow-up duration, SII cutoff values, and survival outcomes including OS, recurrence-free survival (RFS), and cancer-specific survival (CSS). Considering the confounding factors of each study, HRs were extracted from the multivariate analysis.

### Quality Assessment

The Newcastle–Ottawa Scale (NOS) was used to assess the quality of the included studies (16). The NOS includes three parts: patient selection, comparability of research groups, and outcome assessment. The total NOS score ranged from 0 to 9, and studies with scores ≥ 7 were considered to have high quality.

### Statistical Analysis

All data analyses were performed using Stata 16.0 software (Stata Corp, College Station, TX, USA). HRs and 95% CIs were directly extracted from each study. When data could not be extracted, Engauge Digitizer 11.1 software was used to extract survival data from the Kaplan–Meier curves, based on the methods described by Tierney et al. (17).

The heterogeneity of the studies was assessed using the chi-square test with the Higgins I^2^ statistic. If significant heterogeneity existed (I^2^ > 50%), the random-effects model was selected. When heterogeneity was not present (I^2^ < 50%), a fixed-effects model was used (18) . Subgroup analysis were performed based on country, sample size, age, SII cutoff values, treatment method, exclusion of chemotherapy, and the total NOS score of these studies. Metaregression analysis (MRA) was carried out to investigate potential impacts of heterogeneity and confounders on outcomes (19). Factors considered variables included sample size, age, SII cutoff values, and NOS. Begg’s funnel plot, Egger’s funnel plot, and sensitivity analysis were used to assess publication bias. Sensitivity analysis were performed to evaluate the overall results after omitting specific studies. Statistical significance was set at a P-value < 0.05.

## Results

**Figure 1:**
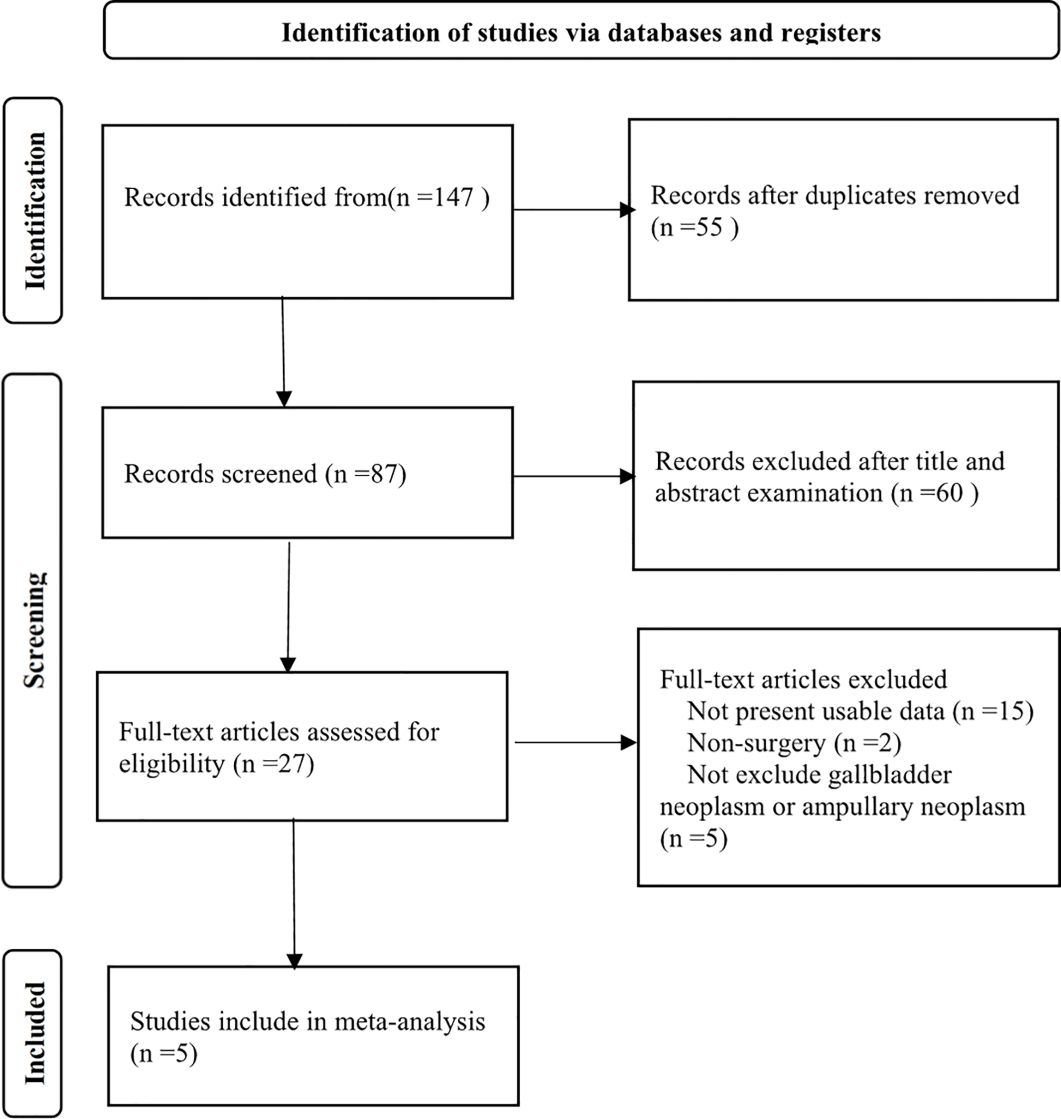
Flow diagram of study selection in the meta-analysis.

The database search process is shown in **Figure 1**. A total of 147 studies were included based on the strategy mentioned previously. Following removal of duplicate studies and initial evaluation by screening titles and abstracts, 27 studies were selected for detailed evaluation. Some studies were excluded for the following reasons: not presenting usable data; nonoperative; and not excluding gallbladder or ampullary neoplasm. Finally, five studies were included in this meta-analysis (20–24) .

The characteristics of these studies are summarized in **Table 1**. The five included studies comprised 1402 patients, ranging from 128 to 688. All studies reported OS, but only two studies reported RFS (21, 22), and one study reported CSS (20). Three studies were conducted in China (21, 22, 24), one in America (20), and one in Japan (23). The cutoff values were not uniform and ranged from 412.6 to 1450. The treatment method of one study included patients who underwent radical or palliative surgery (24), whereas the treatment method used in the other studies was radical surgery. The NOS scores of the included studies are presented in **Table 2**.

**Table 1:** Main characteristic of the included studies.

Author	Year	Study duration	Study design	Sample size	Treatment	SII cutoff values	Outcome	NOS	HR (95%CI)
Tsilimigras DI (20)	2020	2020–2017	Cohort	688	Radical surgery	1150	OS CSS	8	OS: 1.70 (1.23–2.34) CSS: 1.55 (1.09–2.21)
Hui Li (21)	2020	2009–2017	Cohort	530	Radical surgery	450	OS RFS	9	OS: 1.774 (1.245-2.528) RFS: 1.385 (1.005-1.909)
Zeyu Zhang (22)	2020	2013–2017	Cohort	128	Radical surgery	1027	OS RFS	8	OS: 2.454 (1.278-4.712) RFS: 2.368 (1.279-4.386)
Fumihiro Terasaki (23)	2020	2002–2015	Cohort	140	Radical surgery	1450	OS	9	OS: 2.05 (1.03-4.06)
Jian LI (24)	2021	2012–2016	Cohort	181	Radical surgery or palliative surgery	412.6	OS	9	OS: 2.887 (2.256-7.903)

SII, systemic immune-inflammation index; NOS, Newcastle–Ottawa Scale; OS, overall survival; CSS, cancer-specific survival; RFS, recurrence-free survival; HR, hazard ratio; CI, confidence interval.

**Table 2:** Items of NOS of included studies in the meta-analysis.

Study (author year)		Tsilimigras DI 2020	Hui Li 2020	Zeyu Zhang 2020	Fumihiro Terasaki 2020	Jian Li 2021
Selection	Representativeness of the exposed cohort	●	●	●	●	●
	Selection of the non-exposed cohort	●	●	●	●	●
	Ascertainment of exposure	●	●	●	●	●
	Demonstration that the outcome of interest was not present at the start of the study	●	●	●	●	●
Comparability	Comparability of cohorts based on the design or analysis	●○	●●	●○	●●	●●
Outcome	Assessment of outcome	●	●	●	●	●
	Was follow-up long enough for outcomes to occur	●	●	●	●	●
	Adequacy of follow-up of cohorts	●	●	●	●	●
Total score		8	9	8	9	9

### High SII as an Independent Predictor for Poorer OS and RFS

**Figure 2:**
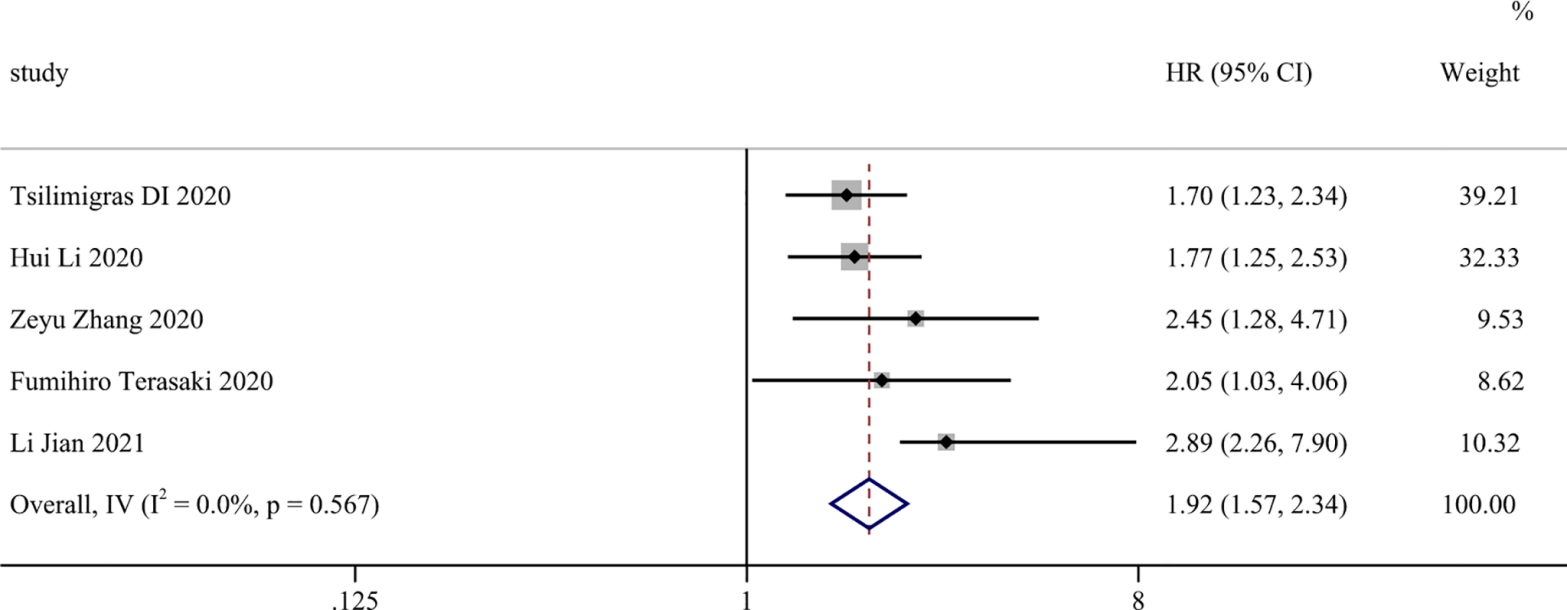
Forest plot of studies evaluating the associations between preoperative SII and OS in cholangiocarcinoma.

**Figure 3:**
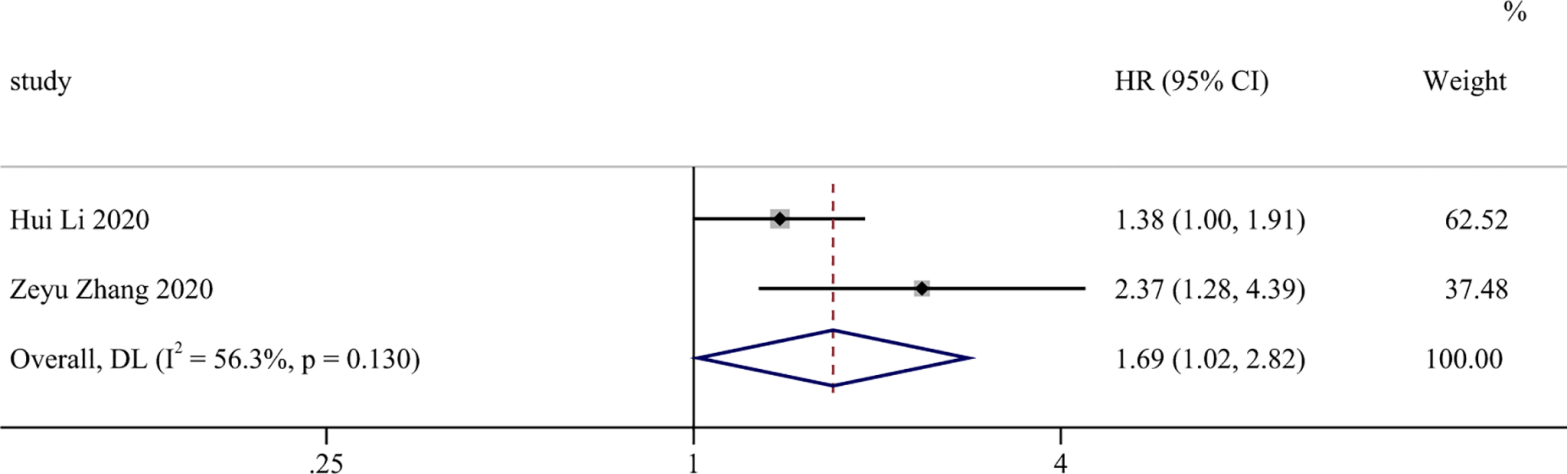
Forest plot of studies evaluating the associations between preoperative SII and RFS in cholangiocarcinoma.

The results of these studies are shown in **Figure 2**. The combined results of the five studies showed the following: HR = 1.916; 95% CI, 1.566–2.343; Z=6.329; and *P* < 0.001. Heterogeneity was not significant; therefore, we used a fixed-effects model (I^2^ = 0.0%). For RFS (**Figure 3**), two studies included in the meta-analysis showed median heterogeneity (I^2^ = 56.3%) with a random-effects model (HR = 1.693; 95% CI, 1.018–2.817; Z = 2.029; *P* = 0.042). The results suggest that an elevated SII was an independent predictor of poorer OS and RFS in patients with CCA.

### Subgroup Analysis and Metaregression Analysis

**Figure 4:**
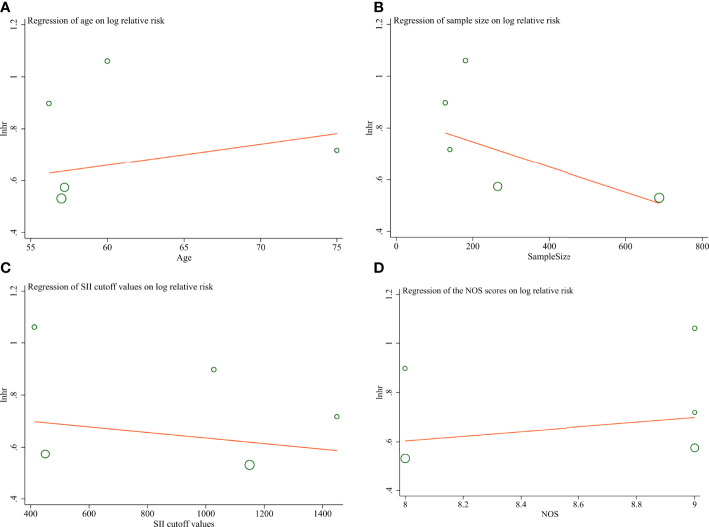
Scattered plots for the metaregression analysis. **(A)** Age **(B)** Sample Size **(C)** SII cutoff values **(D)** NOS.

To detect the prognostic values of preoperative SII in different groups we performed subgroup analysis according to country, age, sample size, SII cutoff values, treatment methods, whether chemotherapy was performed, and the total NOS score of these studies. The subgroup analysis suggested that preoperative SII was still a significant prognostic factor in all subgroups (**Table 3**). To investigate the reasons for heterogeneity of effect, a metaregression analysis was performed. The results of metaregression analysis are graphically shown as scattered plots (**Figures 4A–D**). Metaregression analysis was statistically significant (*P* = 0.025) for sample size in individual studies, whereas age (*P* = 0.896), the SII cutoff values (*P* = 0.064), and NOS (*P* = 0.941) did not explain the demonstrated heterogeneity.

**Table 3:** Subgroup analysis for OS.

Subgroups	No. of studies	Heterogeneity		Fixed-effects model		
		I^2^(%)	*P*	HR (95%CI)	Z	*P*
Total	5	0.0%	0.567	1.92 (1.57–2.34)	6.329	<0.001
Country						
China	3	3.5%	0.355	2.07 (1.57–2.74)	5.124	<0.001
America	1	–	–	1.70 (1.23–2.34)	3.234	<0.001
Japan	1	–	–	2.05 (1.03–4.07)	2.051	0.040
Age (years)						
≥60	2	0.0%	0.567	2.47 (1.56–3.92)	3.831	<0.001
<60	3	0.0%	0.608	1.81 (1.44–2.26)	5.177	<0.001
Sample size						
<150	2	0.0%	0.710	2.25 (1.40–3.61)	3.368	<0.001
>150	3	11.3%	0.324	1.85 (1.48–2.31)	5.409	<0.001
Cutoff value						
<500	2	43.1%	0.185	2.00 (1.47–2.72)	4.393	<0.001
>500	3	0.0%	0.585	1.86 (1.43–2.42)	4.568	<0.001
Treatment						
Radical surgery	4	0.0%	0.774	1.83 (1.48-2.26)	5.558	<0.001
Radical/palliative surgery	1	–	–	2.89 (1.54–5.40)	3.315	0.001
Chemotherapy						
Excluded	3	3.5%	0.539	2.07 (1.57–2.74)	5.124	<0.001
Included	2	0.0%	0.628	1.76 (1.31–2.35)	3.799	<0.001
NOS						
8	2	0.0%	0.323	1.83 (1.37–2.44)	4.093	<0.001
9	3	0.0%	0.414	2.00 (1.51–2.66)	4.848	<0.001

OS, overall survival; HR, hazard ratio; CI, confidence interval.

### Publication Bias

**Figure 5:**
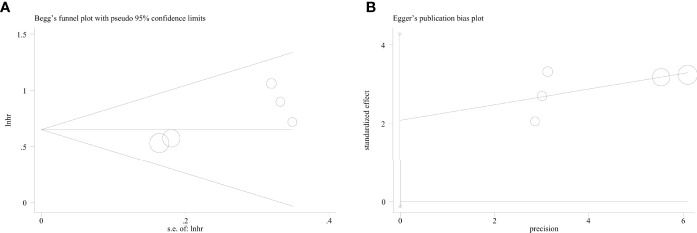
Funnel plot for publication bias in this meta-analysis. **(A)** Begg’s test **(B)** Egger’s test.

**Figure 6:**
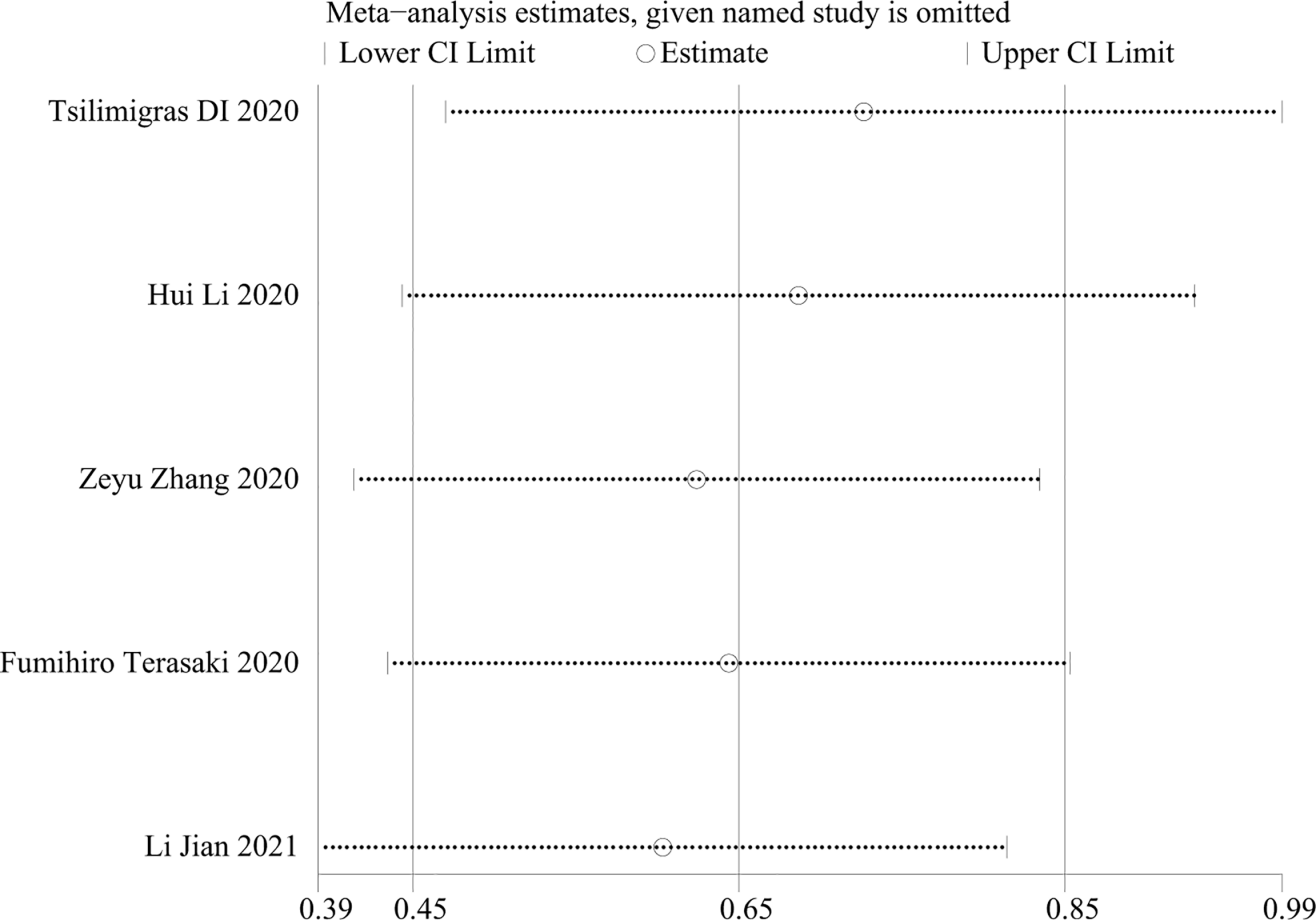
Sensitivity analysis of the studies.

Publication bias was not found in the meta-analysis, as indicated by the symmetry of Begg’s and Egger’s funnel plot (*P* = 0.058). (**Figures 5 A, B**). To prove the stability of the meta-analysis, a sensitivity analysis was performed to determine the effect of the individual studies on the overall conclusion. Excluding any individual of these studies did not change the overall results, confirming the reliability of the meta-analysis (**Figure 6**).

## Discussion

The SII is a composite index based on platelet, neutrophil, and lymphocyte counts. Five studies with 1402 patients were included in this meta-analysis to determine the prognostic value of the preoperative SII. The results showed that a higher SII is associated with poor OS in patients with CCA who have undergone invasive surgery. Moreover, subgroup analysis indicated that country, sample size, cutoff values, treatment methods, whether chemotherapy was performed, and the total NOS score of these studies did not influence the reliability of the results. These findings suggest that a high SII before surgery is a good and powerful predictor of poor survival outcomes in patients with CCA.

The SII is a novel index calculated as follows: SII = platelets × neutrophil/lymphocyte counts; these are routinely used in the clinical setting. SII was first used to predict the prognosis of patients with hepatocellular carcinoma (10). Recently, it has been used to predict the prognosis of other diseases as well. Several studies have shown the prognostic significance of neutrophil-to-lymphocyte ratio (NLR) (25, 26) and platelet-to-lymphocyte ratio (27, 28) in patients with CCA. The mechanisms of the prognostic role of the SII in CCA are unclear, however, current understanding is that tumor-associated systemic inflammatory responses involve various inflammatory mediators and cells (29). Neutrophils, which are the most abundant leukocytes in the body, play diverse roles in immune and cancer processes. The levels of neutrophils can be increased by inflammation or cancer induction. Moreover, neutrophis can participate in tumor initiation, angiogenesis, progression, and metastasis (30, 31). In inflammatory conditions, platelets are activated and interact with endothelial cells and leukocytes. In addition, platelets infiltrate the tumor microenvironment to directly interact with cancer cells and increased platelet counts also increase the risk of venous thrombosis in malignancy. Thrombosis is an adverse complication associated with survival of cancer patients (32, 33). In contrast, lymphocytes play an important role in antitumor immune responses (34). Specifically, they affect tumor growth by secreting cytokines, causing cytotoxic cell death, and preventing the growth and migration of cancer cells (35, 36). Thus, an elevated SII is accompanied by high neutrophil and platelet counts and low lymphocyte counts, and hence can be a good predictor for identifying patients with poor prognosis.

According to the NOS, the scores of the included studies were > 7, which can be considered high quality. The overall sample size was 1402, and the heterogeneity of the meta-analysis was 0.0%, indicating that our results were reliable. All studies included in this meta-analysis were retrospective cohort studies. To ensure the stability and reliability of our results, we used several measures. Considering the confounding factors affecting the results in the survival analysis, the HRs and 95% CIs were extracted from the multivariable analysis. Finally, the fixed-effects model was selected based on the low level of heterogeneity in the results.

In conclusion, our study identified the preoperative SII as a sensitive prognostic factor for OS and RFS in patients with CCA. Given the limited number of studies included in the analysis, more clinical trials and retrospective studies are required in the future.

## Data Availability Statement

The original contributions presented in the study are included in the article/supplementary material. Further inquiries can be directed to the corresponding author.

## Author contributions

XCL and XJ contributed to study conception and design. XCL and JJZ collected the data. XCL, YPJ and XJ analyzed and interpreted the data. XCL wrote the manuscript. YPJ, XGS, LYZ, and XJ made critical revisions to the article. All authors contributed to the article and approved the submitted version.

## Funding

This work was supported by grants from the National Natural Science Foundation of Shandong Province [grant numbers ZR202103040311].

## Conflict of Interest

The authors declare that the research was conducted in the absence of any commercial or financial relationships that could be construed as a potential conflict of interest.

## Publisher’s Note

All claims expressed in this article are solely those of the authors and do not necessarily represent those of their affiliated organizations, or those of the publisher, the editors and the reviewers. Any product that may be evaluated in this article, or claim that may be made by its manufacturer, is not guaranteed or endorsed by the publisher.
